# 

**Published:** 1906-06-23

**Authors:** 


					THE HOSPIT'A'L* " " ? v ' *
* H_W JtrxE 23rd 1903.
o.\ in v
RIVAL
go- 1,031?; Vol. XL. [af^Ser!] SATURDAY,
June 23rd, 1906.
Dscr
see 3HS20.
? THREEPENCE

				

